# The Influence of Different Lay-Up Parameters on the Fatigue Response of Carbon/Epoxy Laminates under Internal Multiaxial Stress States

**DOI:** 10.3390/ma14247494

**Published:** 2021-12-07

**Authors:** Kalliopi-Artemi Kalteremidou, Danny Van Hemelrijck, Lincy Pyl

**Affiliations:** Department of Mechanics of Materials and Constructions, Vrije Universiteit Brussel, Pleinlaan 2, 1050 Brussels, Belgium; Danny.Van.Hemelrijck@vub.be (D.V.H.); Lincy.Pyl@vub.be (L.P.)

**Keywords:** multiaxial fatigue, damage accumulation, fatigue crack growth, S-N curves, unbalanced laminates

## Abstract

The inherent anisotropy of composites complicates their damage response. The influence of multiaxiality, particularly in carbon-based composites, is not thoroughly understood due to obstacles related to damage monitoring during loading. In this study, the response of different carbon/epoxy laminates under fatigue is examined through dedicated in situ microscopic observations. By varying the orientation of off-axis layers, the impact of multiaxiality on the mechanical and damage response is evaluated. Furthermore, balanced and unbalanced laminates are compared, considering the limited information for the latter. The influence of the number of off-axis layers is finally assessed leading to important conclusions about optimal fatigue response. The fatigue response is evaluated in all cases considering both the mechanical properties and the damage characteristics. Significant conclusions are drawn, especially for the benefits of unbalanced laminates and the impact of shear stresses, allowing for the utilization of the obtained data as important input for the establishment of reliable fatigue damage models.

## 1. Introduction

Understanding the behaviour of composite materials under multiaxial dynamic loads is essential in order to design high-performance materials and to develop physically based models suitable to predict the fatigue life and damage. A quite detailed review on the multiaxial fatigue investigation of composite materials was given by Quaresimin [1]. According to this review, multiaxial studies on composites are still limited, and dedicated experimental campaigns have to be performed in order to understand in depth how different multiaxial conditions influence the fatigue damage accumulation. The majority of the fatigue experimental works in composites concern common unidirectional (UD) or cross-ply lay-ups in which nearly uniaxial stress states are developed and the multiaxiality is not taken into account. By contrast, in cases where multiaxiality is indeed considered, damage studies are limited either to damage initiation or to damage propagation; therefore, detailed damage sequences and their correlation with generic stress states are hardly reported [2]. Especially elaborate damage studies in Carbon Fibre Reinforced Polymers (CFRPs) when different multiaxial stress states occur are rarely found.

Indeed, a large experimental library can be found in the literature regarding fatigue testing of UD or cross-ply laminates, in which mainly uniaxial stress states are developed [3,4,5,6,7,8,9,10,11]. The damage response of [45°/−45°] laminates during fatigue has been also investigated [12,13]. However, also in this type of angle-ply laminates, pure shear is developed in the individual plies. In all these cases where more straightforward uniaxial stress states are developed, the initiation and growth of matrix cracks and delaminations has been monitored in detail using different techniques, and the fatigue damage accumulation has been clearly identified [14,15,16,17,18]. It is commonly accepted that damage initiates in the form of matrix cracks which lead to interlaminar delaminations propagating and leading to final failure [19,20,21]. However, it is still not clear how more complex multiaxial stress states influence the fatigue damage initiation and the consequent damage accumulation.

As a matter of fact, experimental studies on the fatigue damage response of polymer composites under multiaxial stresses are rarely found. Flat laminates consisting of off-axis plies have been, for instance, examined in [22,23,24]. However, the fatigue response of the composites in the majority of these cases has been studied mainly in terms of S-N curves [25,26] or stiffness degradation measurements, and detailed damage monitoring is not reported. Damage measurements in laminates under multiaxial loading are very limited in the literature. May et al. [27] examined, for instance, the matrix cracking initiation during fatigue in CFRP [(0°)_2_/(90°)_4_]_s_ and [(0°)_2_/(60°)_4_]_s_ laminates using X-rays and Acoustic Emission (AE). However, detailed damage monitoring throughout the total material life is not reported. Quaresimin et al. [28] compared the damage initiation and propagation in Glass Fibre Reinforced Polymer (GFRP) tubular and flat specimens when the same multiaxial stresses were developed. However, also in this case, only matrix cracking phenomena are reported and correlated with the developing multiaxial stresses. Moreover, their approach is restricted to GFRPs because of the transparency of the glass fibres, allowing for damage monitoring by fixing the proper lightning conditions [29,30,31,32]. Thus, the need to study the fatigue damage response of CFRP laminates under multiaxial stresses has been made clear through the lack of the existing literature in polymer composites.

Multiaxiality in composites can be applied in two ways: internally and externally. The internal multiaxiality in composites arises from their inherent anisotropy. This means that even under simple uniaxial loading of flat laminates, multiaxiality can be developed in off-axis plies, i.e., in plies where the fibres are not parallel to the loading direction. External multiaxiality can be applied using two loading systems: for instance, by performing combined tension/torsion tests in tubes [33,34,35] or by applying biaxial loading to cruciform specimens [36,37,38]. In general, when it comes to studying the fatigue behaviour of composites under multiaxial loads, it should not be necessary to distinguish between internal and external multiaxiality as far as the local stresses in the material remain identical [1]. Tubular specimens have been shown to be quite effective for multiaxial testing of composites, but some influence of the thickness-to-radius ratio on the fatigue life has been reported [39]. Moreover, even though the absence of free edges on tubes can guarantee that no interlaminar stresses are developed, it can also be considered as a significant disadvantage because damage monitoring is restricted. Regarding cruciform specimens, dedicated research has demonstrated that it is quite difficult to obtain an optimised geometry that can guarantee successful biaxial testing by localising the damage in the central part of the specimen. On the other hand, widely applied uniaxial testing of flat specimens gives the possibility to study the multiaxiality in composites just by altering the stacking sequence. Angle-ply laminates can be tested for this purpose, and by selecting the angle θ of off-axis layers, certain biaxial or multiaxial conditions can be established. The free edges of the flat specimens are sometimes considered a drawback due to the nucleation of high interlaminar stresses leading to delaminations. However, in the majority of real applications, flat laminates are used consisting of free edges. Thus, the knowledge of the impact of multiaxial stress conditions on the fatigue damage initiation and growth in such geometries is of great importance.

To that end, an extensive fatigue testing campaign is described in this work. The effect of multiaxiality is primarily investigated. Eight-ply laminates including off-axis layers with an orientation equal to 30° or 60° were tested to establish different multiaxial stress states and to examine the influence of shear on the fatigue behaviour. Furthermore, balanced laminates were compared with unbalanced lay-ups with the same off-axis layers. In a balanced laminate, for every +θ ply, another −θ ply of the same thickness and material exists. This is not the case for the unbalanced laminates used in this study, consisting only of +θ plies. A beneficial response of the unbalanced laminates was identified during a quasistatic study of the considered laminates in a previous work of the authors [40]. The potential of an improved fatigue response is therefore evaluated in the current study. The impact of the number of the off-axis layers is also examined by testing 12-ply laminates, including eight instead of four off-axis plies. Apart from these parameters, the impact of the fatigue stress level as well as the influence of the R-ratio were investigated.

The fatigue response was evaluated considering both the mechanical properties and the damage characteristics. Initially, S-N_f_ (stress-cycles to failure) data for the different types of laminates were established. Their residual strength was then obtained and correlated with the multiaxial stresses. The fatigue damage accumulation was afterward reported. Detailed damage monitoring was performed by attaching an optical microscope on the test bench and by scanning the edge of the specimen at different test intervals while keeping it loaded. The matrix cracking increase and the delamination growth during testing were precisely revealed in order to examine the impact of shear on the damage accumulation, as well as the response of the rarely studied unbalanced laminates. An effort was made to establish empirical rules for the prediction of damage accumulation based on matrix cracking density measurements when different stress components dominate in the off-axis layers. The stiffness degradation and the evolution of the Poisson’s ratio were finally reported for the examined laminates as measured by using a Digital Image Correlation (DIC) system. Significant differences were observed, allowing for the utilisation of the obtained data as an important input for the establishment of reliable fatigue damage models.

## 2. Materials and Methods

### 2.1. Selection of Material

The material used in the present study is TR 360E250S pre-preg CFRP in the form of flat laminates, manufactured by Mitsubishi Chemical Corporation and Honda R&D Co., Ltd. (Tokyo, Japan). The pre-preg consists of PYROFIL #361 130 °C curing-modified epoxy resin and PYROFIL TR 50S15L continuous PAN-based carbon fibres. The material was cured in an autoclave for 60 min at a temperature of 130 °C and a pressure of 0.6 MPa. The mechanical properties of the CFRP composite material were obtained by standard tensile tests on [0°]_4_, [90°]_8_ and [45°/−45°]_2s_ specimens with dimensions according to ASTM D3039 [41] and are summarised in Table 1 (with σ_11,ult_, σ_22,ult_ and τ_12,ult_ representing the ultimate longitudinal, transverse and shear strengths, respectively; E_11_, E_22_ and G_12_ the longitudinal, transverse and shear elastic moduli, respectively; and ν_12_ the Poisson’s ratio, all calculated in the material coordinate system).

As also mentioned in the introduction, the influence of three parameters is studied in this work, i.e., the occurrence of multiaxial stresses, the comparison between balanced and unbalanced lay-ups and the number of off-axis plies. For this reason, six angle-ply flat laminates were tested. Here, [0°/θ]_2s_ unbalanced laminates were initially chosen for two different θ values to account for dissimilar multiaxiality in the off-axis layers. The characteristic of this lay-up is that no negative counterpart of the angle θ exists in the laminate. Secondly, [0°/θ/0°/−θ]_s_ balanced laminates with the same θ values were tested. Finally, [0°/θ/−θ]_2s_ laminates were assessed to examine the influence of the number of off-axis layers on the mechanical response.

**Figure 1:**
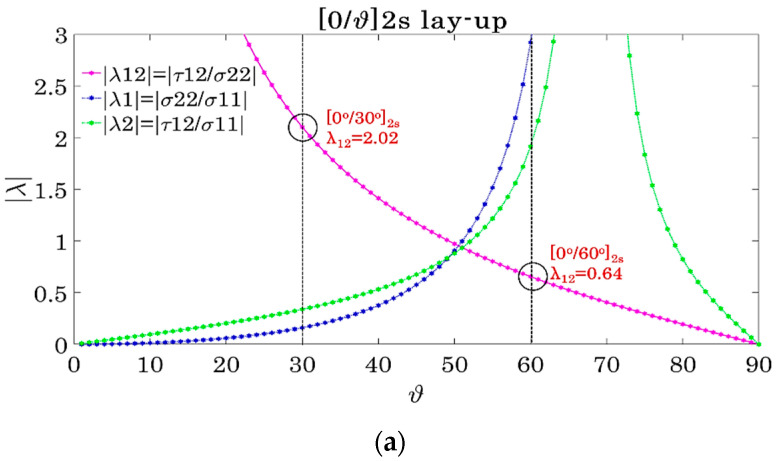

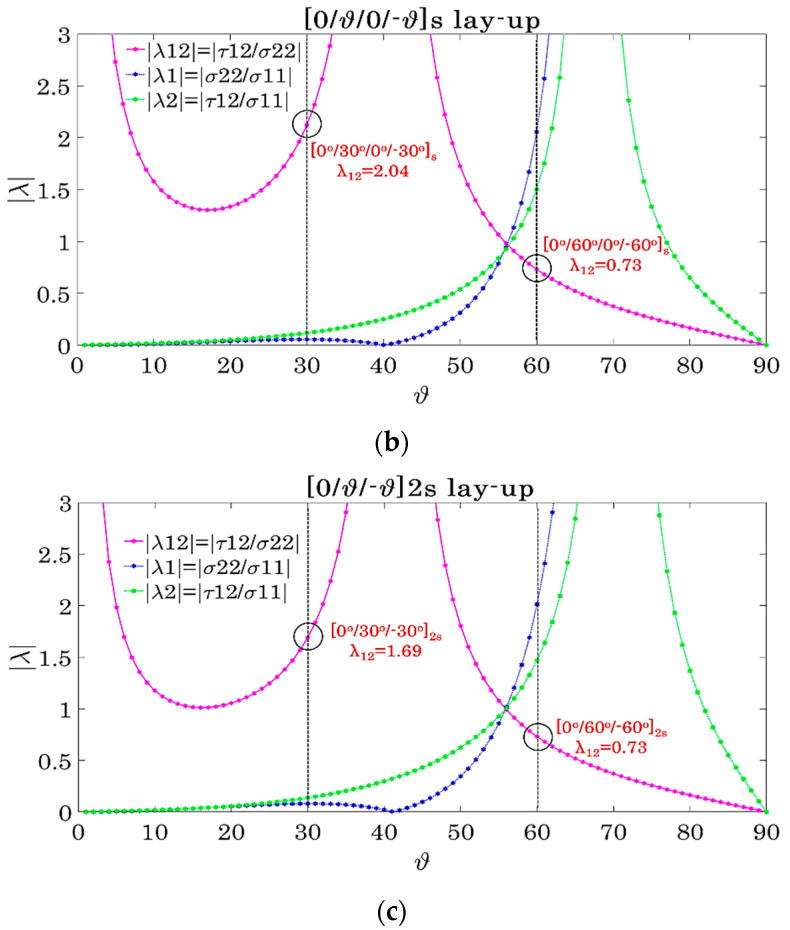
Absolute biaxiality ratios λ in the off-axis layers of (**a**) [0°/θ]_2s_, (**b**) [0°/θ/0°/−θ]_s_ and (**c**) [0°/θ/−θ]_2s_ laminates versus θ.

The off-axis angle values θ were chosen based on calculations using the Classical Laminate Theory (CLT). Considering that most failure criteria make use of stresses and strains in the material coordinate system, it was decided to choose the angle of the off-axis layers based on the biaxiality ratios λ, which express the relation between the normal and shear stresses in the principal directions [1], defined as:

(1) $$\left|\lambda_{1}\right|=\left|\frac{\sigma_{22}}{\sigma_{11}}\right|,\;\left|\lambda_{2}\right|=\left|\frac{\tau_{12}}{\sigma_{11}}\right|,\;\left|\lambda_{12}\right|=\left|\frac{\tau_{12}}{\sigma_{22}}\right|$$

where σ_11_ and σ_22_ are the in-plane longitudinal and transverse stress components and τ_12_ is the in-plane shear stress in the material coordinate system. Special focus was given to the λ_12_ biaxiality ratio, linking the in-plane transverse with the shear stress, to study the influence of their combination on the mechanical response. Based on calculations from the CLT, the evolution of the absolute values of the λ ratios is plotted in Figure 1 versus the angle θ for all considered laminates. To obtain a different multiaxial condition, two angles were chosen based on Figure 1, namely 30° and 60°. It can be seen from Figure 1a that in the [0°/θ]_2s_ lay-up the λ_12_ ratio is equal to 2.02 for θ = 30°, whereas in the 60° layers, it equals 0.64. These values depict that in the [0°/30°]_2s_ laminates, the shear stresses τ_12_ are dominant in the off-axis plies, while the transverse stresses σ_22_ represent the highest stress component in the 60° layers of the [0°/60°]_2s_ laminates. In both cases, the stress along the fibre direction σ_11_ is much lower than the unidirectional strength of the material σ_11,ult_ in the off-axis layers, as depicted by looking at the λ_1_ and λ_2_ ratios in Figure 1a, showing that the σ_11_, σ_22_ and τ_12_ stresses are of the same order of magnitude. Moreover, the same boundary conditions apply for both cases, with the insertion of 0° plies alternating the off-axis layers. This indicates that a direct comparison between the two laminates is allowed, letting the examination of the different matrix-dominated behaviour in the off-axis layers depend on either dominant shear or transverse stresses and not on fibre-related phenomena.

In Figure 1b, the corresponding absolute biaxiality ratios for the balanced laminates are plotted. It can be calculated that λ_12_ equals 2.04 in the 30° plies of the [0°/30°/0°/−30°]_s_ laminates and 0.73 in the 60° layers of the [0°/60°/0°/−60°]_s_ laminates. Finally, to examine the influence of the relative number of the off-axis layers θ with respect to the number of 0° layers, [0°/30°/−30°]_2s_ and [0°/60°/−60°]_2s_ laminates were tested, consisting of eight instead of four off-axis plies. In Figure 1c, the variation of the absolute biaxiality ratios for the thicker 12-ply [0°/θ/−θ]_2s_ lay-up is plotted, with λ_12_ equalling 1.69 in the 30° plies of the [0°/30°/−30°]_2s_ laminates. This shows that by increasing the number of the off-axis plies, λ_12_ decreases; thus, the shear influence has the tendency to decrease. However, the behaviour remains shear-dominated. This phenomenon diminishes by increasing θ. In the 60° off-axis layers of the [0°/60°/−60°]_2s_ laminates, λ_12_ equals 0.73, being similar to the 8-ply [0°/60°]_2s_ and [0°/60°/0°/−60°]_s_ laminates.

**Figure 2:**
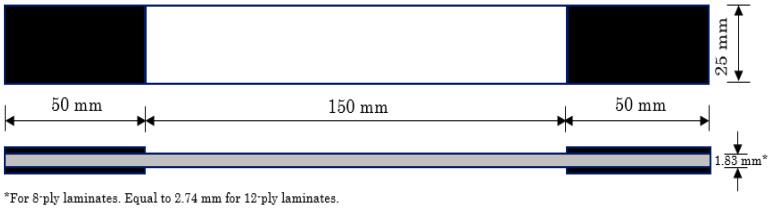
Specimen dimensions.

The dimensions of the tested specimens were defined based on [41]. All samples had a total length equal to 250 mm and a width of 25 mm. The thickness of the 8-ply laminates was equal to 1.83 mm on average, whereas for the 12-ply laminates, it equalled 2.74 mm (Figure 2). All specimens were tabbed for a length of 50 mm on both sides using GFRP material in a [0°/90°]_2s_ configuration to ensure failure within the gauge length.

### 2.2. Experimental Details

An MTS fatigue testing system with two servohydraulic grips, with a maximum load capacity of 100 kN, was used for the experimental campaign. Constant amplitude load-controlled fatigue tests at a frequency of 3 Hz were performed for all considered laminates. Initially, continuous fatigue loading was applied to all specimens until catastrophic failure occurred or until the fixed run-out value was reached. Three maximum fatigue stress levels σ_max_ were applied, corresponding to 70%, 80% and 90% of the respective ultimate strength σ_ult_ for each laminate. Σ_ult_ was obtained from initial static tests, and it was found to be equal to 1375, 1318, 1057, 1213, 967 and 891 MPa for the [0°/30°]_2s_, [0°/60°]_2s_, [0°/30°/0°/−30°]_s_, [0°/60°/0°/−60°]_s_, [0°/30°/−30°]_2s_ and [0°/60°/−60°]_2s_ laminates, respectively [40]. One more stress level with σ_max_ equalling 60% of σ_ult_ was applied for the continuous tests to verify the endurance limit of all laminates. Two R-ratios were considered, namely 0.1 and 0.5. The run-out value was set to one million cycles, typical for fatigue studies in composites.

Based on the continuous tests and the total number of cycles to failure N_f_ for each case, interrupted fatigue tests were then performed to assess the microscopic through-thickness damage evolution at certain fatigue intervals for the 70%, 80% and 90% test cases. For the specimens that reached the run-out value of one million cycles during the continuous tests, test interruptions were performed after 10, 50, 200, 10^3^, 10^4^, 10^5^, 3 × 10^5^, 5 × 10^5^, 7.5 × 10^5^ and 10^6^ cycles. The same initial intervals were applied to the rest of the specimens as well, but the frequency of microscopic monitoring was altered based on the obtained N_f_ from the continuous tests for each case. The frequency of microscopic inspection is given in Table 2.

**Figure 3:**
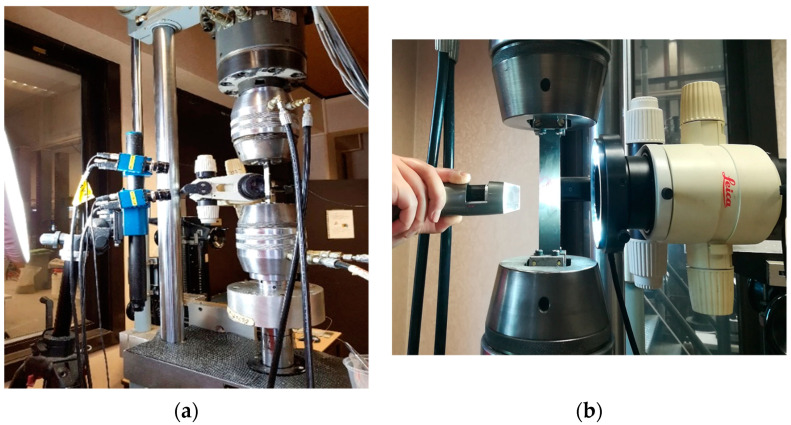
(**a**) Experimental set-up and (**b**) microscopes for damage monitoring.

An optical microscope was used for in situ through-thickness monitoring of the fracture patterns during the interrupted tests. Specifically, each time a specific number of cycles during the fatigue tests was reached (according to Table 2), the tests were interrupted, and the specimens were kept under a load close to the maximum fatigue load applied at each case. By keeping the specimens tensioned, through-thickness damage observations on their free edge were performed along their gauge length. This was possible by attaching special bars on the test bench, which allowed the movement of the optical microscope along the length of the specimens (Figure 3). By scanning the total length of the specimens, the measurement of the matrix crack densities and the delamination lengths was possible using the corresponding measurement tools of the microscope’s software. The advantage of this set-up is that load is still applied to the specimens during the observations; therefore, the occurring cracks are still open and visible, in contrast to the cases in which only postmonitoring is performed. The microscope used was an MZ125 stereomicroscope by Leica Microsystems with 8–100× magnification, attached to a Leica DFC 295 digital camera with a standard resolution of 3 MP. Prior to testing, the free edges of the samples were polished starting with fine SiC grit papers of 46 µm particle size and going down to 5 µm. This eliminated weak areas at the free edges whilst allowing for recording sharp images. In order to confirm that the findings of the microscope along one edge of the specimens were not caused by an unbalanced load introduction, a Dino-Lite USB microscope with a 1.3 MP sensor and 20–230× magnification was used for damage monitoring on both sides of the specimens, during all test intervals. Even if optimal focus is not achieved with this microscope, since it is not mounted on the test bench, the advantage is its flexibility as it can be used to scan the total length of the specimens.

A DIC system was also utilised during the fatigue tests for strain measurements. Two Charge Couple Device (CCD) cameras were mounted in front of the testing samples, and a random speckle pattern was applied to their front side. By triangulation between the two cameras, 3D displacement and strain measurements on their front surface could be provided. Two 23 mm lenses were used to capture the DIC images. A subset of 21 pixels by 21 pixels was used, and a step of 7 pixels was applied during the DIC analysis, performed with the VIC-3D software of Correlated Solutions. The features of the DIC system are shown in Table 3, including the obtained resolution and sensitivity. The DIC system was fixed to take one image during the application of the maximum and minimum load (peaks) to the sample at specific cycle points. The image acquisition frequency through the DIC system is shown in Table 4. Apart from the DIC system, an MTS extensometer with 50 mm gauge length and 5% measuring strain range was used for strain measurements on the opposite side of the specimens during all examined test cases. In Figure 3a, the experimental set-up is presented, showing the exact placement of the measuring systems. One indicative image of the applied microscopes on the MTS test bench is shown in Figure 3b.

## 3. Results and Discussion

### 3.1. S-N_f_ Data

**Figure 4:**
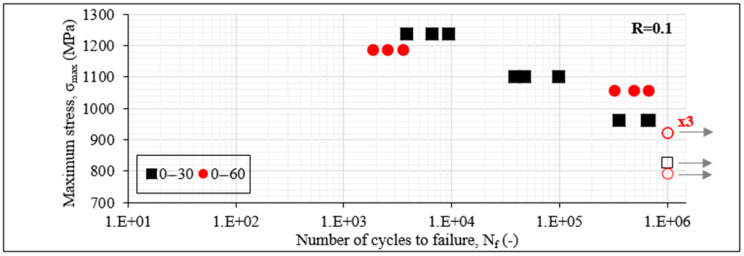
S-N_f_ data of [0°/30°]_2s_ and [0°/60°]_2s_ laminates for R = 0.1.

The initial information derived from any fatigue experimental campaign is the S-N_f_ data. In Figure 4, the S-N_f_ data for the unbalanced laminates are initially plotted for R = 0.1. On the *x*-axis, the cycles to failure N_f_ are plotted on a logarithmic scale. On the *y*-axis, the σ_max_ values are plotted on a linear scale. The filled data points mean that N_f_ was less than 10^6^ cycles, whereas the unfilled ones indicate run-outs, meaning that the corresponding specimen reached the run-out limit of one million cycles. The grey arrows also designate the same for clarity. Keeping in mind the generally stochastic response of composite materials under fatigue loads, the obtained results present a good repeatability, with data points corresponding to the same fatigue conditions being always in the same order of magnitude. It can be noticed from Figure 4 that the [0°/60°]_2s_ laminates lead to a generally better fatigue behaviour in terms of the cycles to final failure than the [0°/30°]_2s_ laminates. Run-outs are observed in both cases when the maximum fatigue stress is equal to 60% of σ_ult_. This can be explained by the fact that carbon/epoxy laminates are quite fatigue resistant. This means that any difference in the fatigue behaviour is revealed only at high fatigue loads, explaining thus the choice of the high stress levels for the fatigue experimental campaign. It can be seen from Figure 4 that when R = 0.1, the [0°/60°]_2s_ laminates lead to run-out even for σ_max_ equal to 70% of σ_ult_, whereas this is not the case for the [0°/30°]_2s_ laminates which fail after 3–7 × 10^5^ cycles. This difference is also obvious when the maximum fatigue stress equals 80% of σ_ult_, with the [0°/30°]_2s_ laminates failing after 3–9 × 10^4^ cycles, whereas N_f_ is in the order of 10^5^ cycles for the [0°/60°]_2s_ laminates. This behaviour is inversed for the very high fatigue loads with σ_max_ equalling 90% of σ_ult_. In this case, both laminates lead to fatigue lives of some thousands of cycles, with the [0°/60°]_2s_ laminates being characterised by relatively fewer cycles to failure.

As a first conclusion, it can be assumed that the high shear stresses in the off-axis layers of the [0°/30°]_2s_ laminates lead to quicker degradation of the material and consequently to lower fatigue life. This is confirmed later by the detailed damage investigations. The oppositeness for the very high fatigue loads can be attributed to the high matrix crack densities occurring in the [0°/60°]_2s_ laminates, leading in this case more quickly to catastrophic failure, however without significant differences in the N_f_ values.

**Figure 5:**
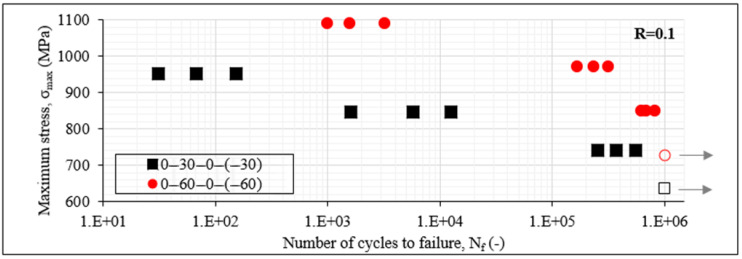
S-N_f_ data of [0°/30°/0°/−30°]_s_ and [0°/60°/0°/−60°]_s_ laminates for R = 0.1.

**Figure 6:**
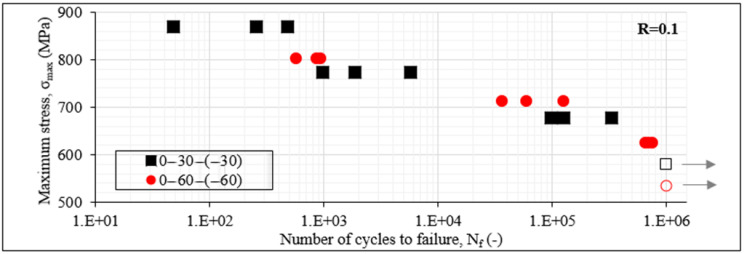
S-N_f_ data of [0°/30°/−30°]_2s_ and [0°/60°/−60°]_2s_ laminates for R = 0.1.

To confirm the previous results, in Figure 5, the S-N_f_ data for the [0°/θ/0°/−θ]_s_ laminates for both angles and for R equal to 0.1 are plotted. In this case, the influence of the different multiaxial stresses is much more obvious. The balanced [0°/60°/0°/−60°]_s_ laminates lead to significantly better fatigue response than the [0°/30°/0°/−30°]_s_ laminates. Both lead to run-out when σ_max_ equals 60% of σ_ult_. The [0°/60°/0°/−60°]_s_ laminates lead then always to higher fatigue life for all stress levels, even if σ_max_ is similar in both cases. This is also confirmed from Figure 6 plotting the S-N_f_ data for the [0°/θ/−θ]_2s_ laminates. In addition, in this case, the shear-dominated [0°/30°/−30°]_2s_ laminates are characterised by lower fatigue lives than the [0°/60°/−60°]_2s_ laminates in which the transverse stresses dominate in the off-axis layers. Apart from the 60% maximum fatigue stress case, at which both laminates lead to run-out, for the rest of the stress levels, the [0°/60°/−60°]_2s_ laminates fail always after a higher number of cycles. A characteristic example is that for σ_max_ equal to 70% of σ_ult_, the [0°/60°/−60°]_2s_ laminates fail after 6–7 × 10^5^ cycles, whereas the [0°/30°/−30°]_2s_ laminates have a fatigue life in the range of only 1–3 × 10^5^ cycles, which means that their fatigue life is less than half of the [0°/60°/−60°]_2s_ laminates.

Comparing the unbalanced with the balanced laminates in terms of their fatigue life in Figure 4 and Figure 5, the potential of the first ones is revealed. For all stress cases, the unbalanced laminates fail after a higher number of cycles. Indicatively, for the 90% case, a big difference appears between the [0°/30°]_2s_ and [0°/30°/0°/−30°]_s_ laminates, with the first ones failing after some thousands of cycles, while the latter ones only survive for some dozens of cycles. These findings agree with the results obtained after the analysis of static tests [40], showing that unbalanced laminates can perform better than balanced ones, delaying the onset and propagation of damage.

Trying to explain the influence of the number of the off-axis layers on the fatigue response of the material by comparing Figure 5 and Figure 6, the impact is more obvious for the 60° angle case. If the 8-ply [0°/60°/0°/−60°]_s_ laminates are compared with the 12-ply [0°/60°/−60°]_2s_ laminates, it is obvious that the last ones have a worse fatigue behaviour, leading to three times lower fatigue lives on average for the 80% and 90% stress cases. The influence is smaller for the 30° off-axis angle, with the [0°/30°/−30°]_2s_ laminates leading to similar fatigue lives with the [0°/30°/0°/−30°]_s_ laminates. This could be related to the multiple interfaces in the 12-ply lay-up which accumulate the damage at independent regions without crucial propagation, as is discussed later.

**Figure 7:**
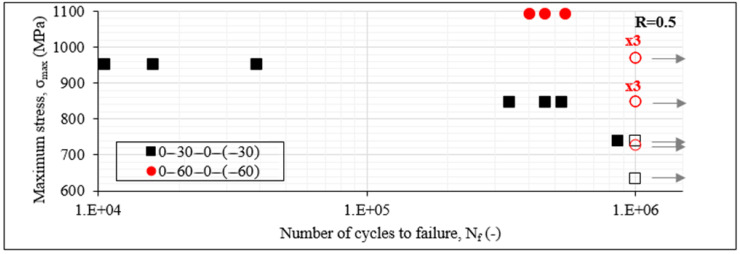
S-N_f_ data of [0°/30°/0°/−30°]_s_ and [0°/60°/0°/−60°]_s_ laminates for R = 0.5.

The previous results related to the impact of shear, the comparison between unbalanced and balanced laminates and the influence of the number of the off-axis layers were confirmed for an R-ratio equal to 0.5 as well. In all cases, the R-ratio seemed to have a great impact on the fatigue life, with higher fatigue lives being registered for all laminates and for all considered stress cases for an R-ratio equal to 0.5, corresponding to a lower stress amplitude during the fatigue loading. This impact is indicatively presented in Figure 7, plotting the S-N_f_ data for the [0°/θ/0°/−θ]_s_ laminates for R = 0.5. Both laminates are characterised by much higher fatigue lives when the R-ratio is increased (compared to Figure 5). As an example, the fatigue life of the [0°/30°/0°/−30°]_s_ laminates increases by 64 times when σ_max_ equals 80% of σ_ult_, just by increasing the R-ratio from 0.1 to 0.5.

### 3.2. Residual Strength Measurements

**Figure 8:**
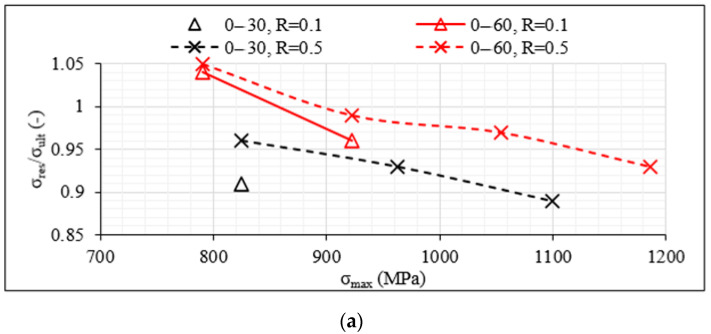

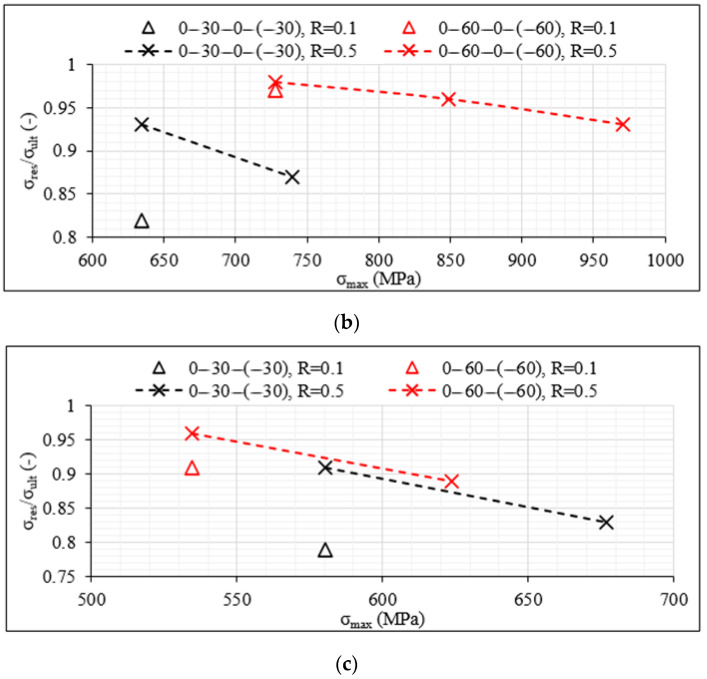
σ_res_/σ_ult_ values versus σ_max_ obtained for (**a**) [0°/θ]_2s_, (**b**) [0°/θ/0°/−θ]_s_ and (**c**) [0°/θ/−θ]_2s_ laminates.

Specimens that survived the fatigue experiment of 10^6^ cycles were subjected to static tensile tests with a displacement rate of 1 mm/min to calculate their residual strength σ_res_. In Figure 8a–c, the average ratio σ_res_/σ_ult_ versus the maximum fatigue stress σ_max_ for both R-ratios, 0.1 and 0.5, is plotted, respectively, for the unbalanced [0°/θ]_2s_, the 8-ply balanced [0°/θ/0°/−θ]_s_ and the 12-ply [0°/θ/−θ]_2s_ specimens that reached the run-out value. By looking first at Figure 8a, the shear influence is once more evident. The [0°/30°]_2s_ laminates lead to a notably higher drop in their final strength compared to the [0°/60°]_2s_ laminates after having survived one million fatigue cycles. The residual strength becomes smaller when the maximum fatigue stress increases, and a drop even higher than 10% is observed for the [0°/30°]_2s_ laminates when σ_max_ reaches 90% of σ_ult_ and R = 0.5. The influence of the R-ratio is also clear and quite detrimental. When R = 0.1, the drop of 10% in strength is observed when σ_max_ equals only 60% of σ_ult_, i.e., during the application of quite lower fatigue loads. On the other hand, in the [0°/60°]_2s_ laminates, higher σ_res_/σ_ult_ values are observed. It is remarkable that even an increase in the final strength is obtained when σ_max_ equals 60% of σ_ult_ for both values of R. This can be attributed to the limited extent of damage in the unbalanced [0°/60°]_2s_ laminates under such low loads together with a potential re-alignment of the 60° layers, resulting in a lower inclination with respect to the loading direction. The impact of the R-ratio and the increasing σ_max_ values on the residual strength is nevertheless the same for both unbalanced laminates.

By looking at Figure 8b,c, the shear influence is also confirmed for the balanced 8-ply and 12-ply laminates, with specimens including 30° off-axis layers being characterised by higher drops in σ_res_. The same applies for the impact of the R-ratio with lower values, related to higher fatigue amplitude, leading always to a higher reduction of the final strength of the laminates after one million cycles. All previously discussed observations regarding the higher fatigue resistance of the unbalanced laminates are confirmed when the residual strength is studied. In all cases and for both off-axis angles, the drop in final strength is always higher for the balanced laminates. A characteristic example can be seen for the case of σ_max_ equalling 60% of σ_ult_. The [0°/30°]_2s_ laminates are characterised by 9% drop in strength for R = 0.1, whereas for the same maximum fatigue stress and the same R-ratio, the corresponding drop for the [0°/30°/0°/−30°]_s_ laminates is double, namely 18%. Similar conclusions are drawn when examining the impact of the number of off-axis layers. Indicatively, for σ_max_ equalling 60% of σ_ult_ and R = 0.1, the σ_res_/σ_ult_ value equals 0.91 for the 12-ply [0°/60°/−60°]_2s_ laminates, whereas it is equal to 0.97 for the 8-ply [0°/60°/0°/−60°]_s_ laminates. It should be highlighted that for the same stress conditions, this ratio equals 1.04 for the unbalanced [0°/60°]_2s_ lay-up.

### 3.3. Investigation of Damage Accumulation

After a thorough experimental campaign of the considered laminates under quasistatic loading [40], it was revealed that matrix cracking is not necessarily the primary damage mode occurring in CFRPs when dominant shear stresses are developed. However, it is well known that matrix cracking is the initial damage mode occurring in composite materials during fatigue. Nevertheless, the onset of matrix cracking and the initiation of consequent interlaminar delaminations depend on many parameters, including the ones examined in this study.

#### 3.3.1. Damage Accumulation in Laminates with 60° Layers

**Figure 9:**
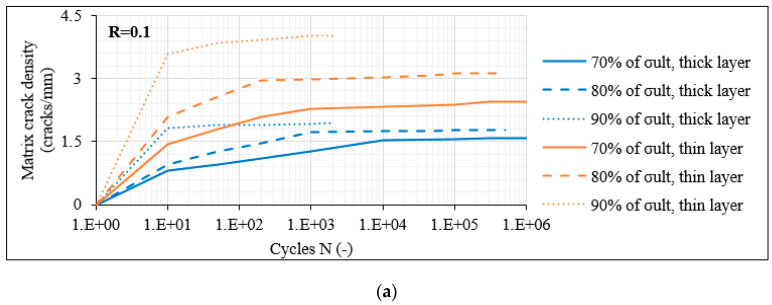

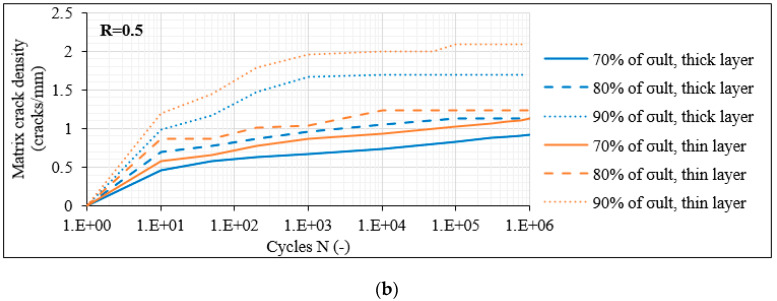
Matrix crack density versus N in [0°/60°]_2s_ laminates for (**a**) R = 0.1 and (**b**) R = 0.5.

The analysis starts with the laminates with 60° layers, in which the domination of the transverse stresses triggers more directly the initiation of matrix cracks. In these laminates, matrix cracking of all off-axis layers along the total gauge length was always the primary damage mode occurring during fatigue loading. In Figure 9a, the average evolution of the measured matrix crack density versus the number of cycles N (on a logarithmic scale) is plotted for the unbalanced [0°/60°]_2s_ laminates for R = 0.1 and for the three maximum fatigue stress levels (i.e., 70%, 80% and 90% of σ_ult_). A distinction between the cracks in the thick layer (middle pair of 60° layers), represented by the blue curves, and the thin single 60° layers, corresponding to the orange lines, is made. It is obvious that matrix cracks initiate with a high rate from the first cycles saturating as the test continues. Increasing the fatigue stress level leads to higher matrix crack density, and greater densities are observed in the thin off-axis plies. It is also notable that the higher the σ_max_, the quicker the crack saturation occurs, whereas the rate is smaller for lower stress levels. In order to reveal the impact of the R-ratio, in Figure 9b, the corresponding results for R = 0.5 are shown. It is obvious that significantly lower crack density values are observed due to the lower stress amplitude related to the higher R-ratio. Despite the fact that in this case as well the crack density shows an increasing trend when σ_max_ increases, the densities measured in the thick and thin plies are much closer at the same stress level. Moreover, it should be mentioned that the cracking rate is lower when R = 0.5 and that saturation does not even occur in some cases, especially for the lower stress levels.

**Figure 10:**
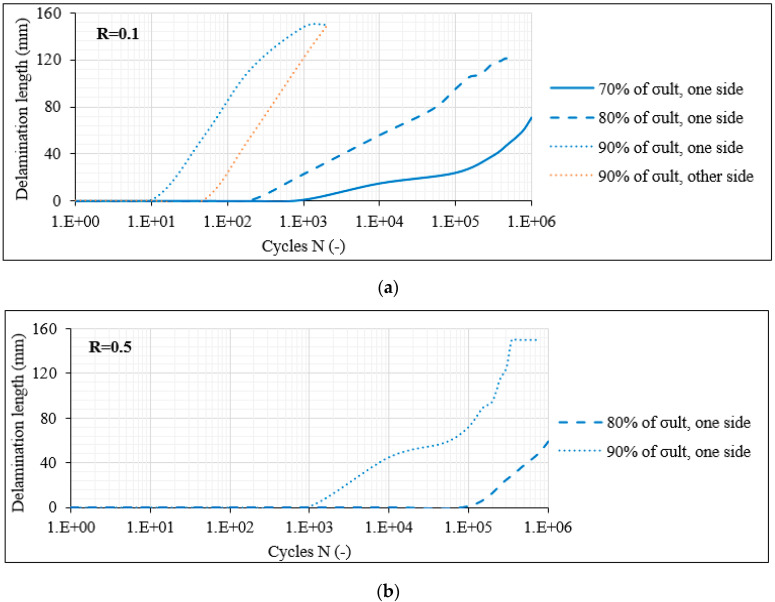
Delamination crack growth versus N in [0°/60°]_2s_ laminates for (**a**) R = 0.1 and (**b**) R = 0.5.

To demonstrate the delamination behaviour in the [0°/60°]_2s_ laminates, in Figure 10a the average delamination crack growth versus N is plotted for R = 0.1. A differentiation between the delaminations along the interfaces of the outer 0° layers and the adjacent 60° layers at both sides of the laminate is performed (blue and orange curves). It is apparent that the initiation of delaminations is delayed and the crack growth rate is decreased when the stress level drops. As an example, when σ_max_ equals 80% of σ_ult_, the first delamination appears after 200–10^3^ fatigue cycles on average, whereas this occurs only after 10^3^–10^4^ cycles for σ_max_ equalling 70% of σ_ult_. Moreover, while delaminations do appear at all examined stress levels, only in the case of σ_max_ equalling 90% of σ_ult_ is a second delamination on the opposite side of the laminate also evident. In Figure 10b, the corresponding results for R = 0.5 are shown. The influence of the R-ratio is evident. For the same stress level, delaminations appear much later in the fatigue life of the laminate when R = 0.5. A characteristic example is that for σ_max_ equal to 80% of σ_ult_, the first delamination appears after 200–10^3^ cycles for R = 0.1 and only after 10^5^ cycles when R = 0.5. For the 70% stress level case, no delamination appeared during the whole duration of the fatigue test with R = 0.5. Moreover, no delamination at the other laminate side was monitored for all stress levels. These trends related to the influence of the R-ratio were confirmed for all laminates under consideration.

Based on the damage observations on the [0°/60°]_2s_ laminates, it can be concluded that predictions for the initiation of interlaminar delaminations can be accomplished, based on measurements of the matrix crack density in the off-axis layers. Stemming from empirical observations, it was noticed that for all fatigue testing conditions, delaminations appeared when the matrix crack density in the off-axis layers reached a saturation level or else when the Critical Damage State (CDS) was exceeded. This observation is further confirmed by looking at the damage state of the [0°/60°]_2s_ laminates for σ_max_ = 0.7 × σ_ult_ and R = 0.5. It is obvious from Figure 10b that no delamination appeared during these testing conditions. Furthermore, it is evident from Figure 9b that the matrix cracks in the off-axis layers did not saturate until the end of the test, justifying the absence of delaminations until the run-out limit.

**Figure 11:**
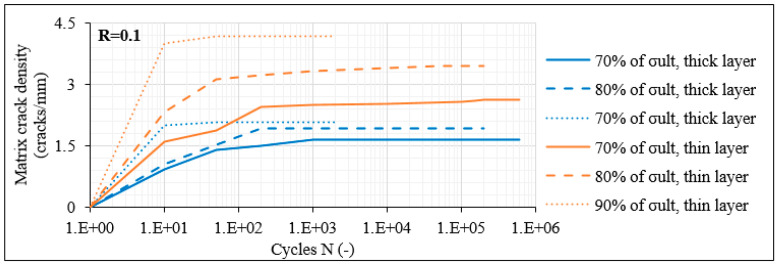
Matrix crack density versus N in [0°/60°/0°/−60°]_s_ laminates for R = 0.1.

**Figure 12:**
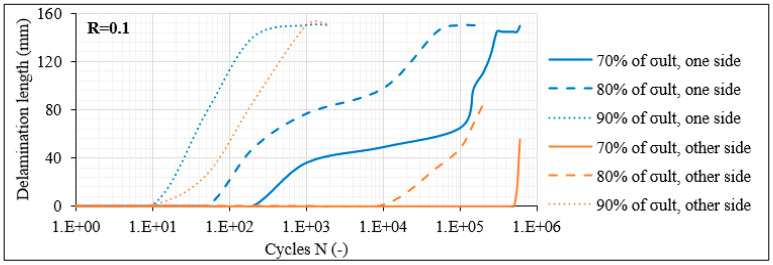
Delamination crack growth versus N in [0°/60°/0°/−60°]_s_ laminates for R = 0.1.

In order to compare the fatigue damage response of the unbalanced with the balanced laminates with 60° layers, the average matrix crack density measured for the [0°/60°/0°/−60°]_s_ laminates for R = 0.1 is plotted in Figure 11. It is clear that both the unbalanced and the balanced laminates result in a very similar fracture process with the initiation of matrix cracks at the very early fatigue cycles and the subsequent initiation and propagation of delaminations. As a first remark, it can be seen that the crack density in the [0°/60°/0°/−60°]_s_ laminates reaches very similar values as in the [0°/60°]_2s_ laminates for the same stress level, with slightly higher values recorded in the balanced laminates, which always increase when the maximum fatigue stress elevates. Moreover, constantly higher values are reported also in this case for the thin layers, compared to the thick ones. Nevertheless, one important difference is observed when comparing the crack densities between the balanced and the unbalanced laminates. It is clear from Figure 9a and Figure 11 that for the same σ_max_ level, the saturation of the matrix cracks occurs earlier in the balanced laminates, meaning that the crack rate is significantly higher in this case. As an example, for the 70% σ_max_ case, the saturation of the cracks occurs after 10^4^ and 10^3^ cycles for the thick and thin layers of the [0°/60°]_2s_ laminates, respectively, whereas the same phenomenon takes place already after 10^3^ and 200 cycles on average in the [0°/60°/0°/−60°]_s_ laminates. This is quite significant since, as discussed previously, the saturation of the cracks is directly linked to the initiation and propagation of interlaminar delaminations. Indeed, also in the case of the balanced laminates, the saturation criterion applies. In all cases, after the matrix cracks in the off-axis layers reached a plateau, interlaminar delaminations between the outer 0° layer and the adjacent 60° layer were nucleated shortly after. Specifically, in Figure 12, the delamination crack growth versus N curves for R = 0.1 are plotted. Delaminations initiate earlier than in the unbalanced lay-up for the same stress level. Moreover, the extent of damage is more detrimental in the [0°/60°/0°/−60°]_s_ laminates. Higher delaminated lengths are measured with two outer-ply delaminations in most of the cases, even in test conditions at which no delamination appeared in the [0°/60°]_2s_ laminates.

**Figure 13:**
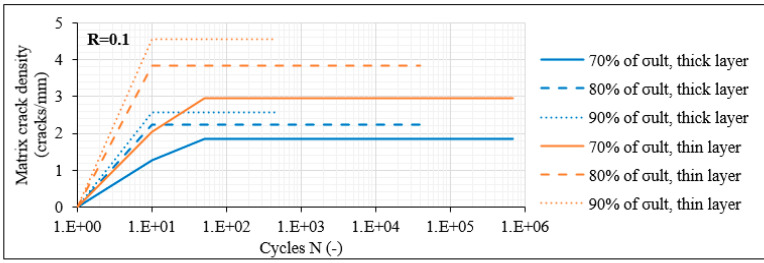
Matrix crack density versus N in [0°/60°/−60°]_2s_ laminates for R = 0.1.

**Figure 14:**
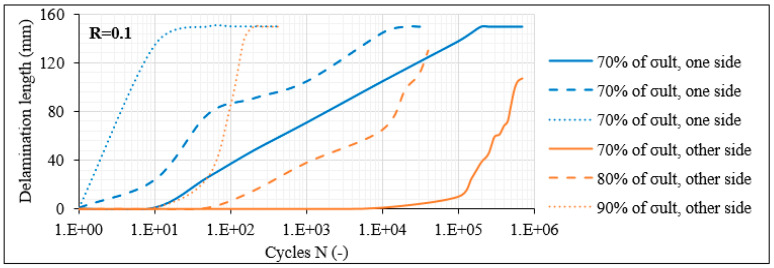
Delamination crack growth versus N in [0°/60°/−60°]_2s_ laminates for R = 0.1.

In order to demonstrate the influence of the number of the off-axis plies, Figure 13 and Figure 14 plot the matrix crack density and the delamination crack growth as measured for the [0°/60°/−60°]_2s_ laminates for R = 0.1. It should be stated here that in the case of the 12-ply laminates, the term “thick” corresponds to the middle double (−θ)/(−θ) layer whereas the term “thin” corresponds to the rest of the layers having no identical lamina next to them. Before describing the influence of the number of the off-axis layers on the fatigue response for θ = 60°, it should be mentioned that certain similarities with the previously studied laminates also having an angle of 60° are obtained. These similarities concern mainly the influence of σ_max_ and R-ratio. Despite these similarities, increasing the number of the off-axis layers in the laminate results in notably higher matrix crack densities in all off-axis layers.

This can be related to a significantly lower fracture toughness of the 12-ply lay-up when off-axis layers with dominant σ_22_ stresses are added. This is also evident by the fact that in this case a quite unstable fracture process was observed. While the aforementioned crack densities correspond to average values along the total gauge length, it was noticed that some laminate areas were much more deteriorated than others, with higher matrix crack densities, and that in some cases, cracks that had not propagated along the total layer thickness appeared. This was not the case in the 8-ply laminates, in which quite stable density distributions were observed with cracks covering the total lamina thickness. Moreover, it is remarkable that the crack density saturates rapidly in the 12-ply laminates, corresponding to a very high cracking rate. Indicatively for R = 0.1 and for all stress levels, the matrix crack density in all off-axis layers saturates after 50 fatigue cycles at the latest. Once again, this is really detrimental since also in this case the saturation of matrix cracks leads quickly to delaminations between the outer plies of the laminate. Regarding the delaminations in the [0°/60°/−60°]_2s_ laminates, apart from their initiation period which appears significantly earlier, the extent of damage is also more detrimental compared to the 8-ply laminates. The delamination growth rate is significantly high, especially for rising stress levels, leading to fully delaminated interfaces quickly during the fatigue test.

#### 3.3.2. Damage Accumulation in Laminates with 30° Layers

To examine the shear influence on the fatigue damage of the composite material, the damage investigation in the [0°/30°]_2s_ laminates is initially reported in the following. It was observed that the high shear stresses τ_12_ in the off-axis layers of the laminates had a detrimental influence especially regarding the initiation and propagation of interlaminar delaminations. Matrix cracking was also in this case the primary damage mode that occurred during the fatigue loading. Nevertheless, both the initiation period and the fracture process were different compared to the case of the [0°/60°]_2s_ laminates. Specifically, matrix cracks were initially observed along the total length of the middle thick layer of the laminate, consisting of the off-axis 30° layers pair. After a certain increase in the matrix crack density, interlaminar delaminations were observed between the outer 0° layer and the adjacent 30° layer, prior to the initiation of matrix cracks in the thin 30° layers. This is apparently contradictory to the results obtained for the [0°/60°]_2s_ laminates. The propagation of delaminations along the 0°/30° interface resulted in the occurrence of cracks in the thin off-axis layers.

**Figure 15:**
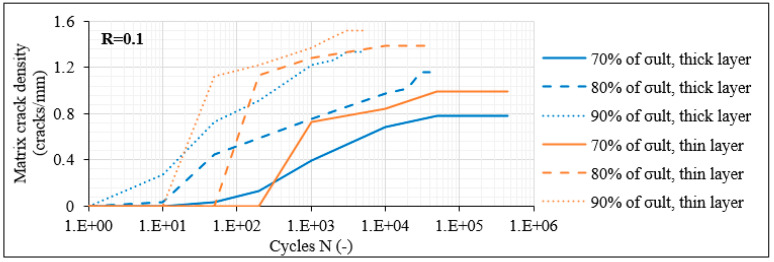
Matrix crack density versus N in [0°/30°]_2s_ laminates for R = 0.1.

In Figure 15, the average matrix crack density evolution versus N both for the thick and the thin off-axis layers is displayed for R = 0.1. It is obvious that for every stress level, matrix cracks initiate first in the middle thick layer, and the thin layers follow. Moreover, matrix cracks in the [0°/30°]_2s_ laminates do not necessarily initiate during the application of the very first fatigue cycles. As an example, while matrix cracks are monitored after 10 fatigue cycles at the 90% stress level case, they are evident after 50 cycles when σ_max_ equals 70% of σ_ult_. The higher the maximum fatigue stress applied, the sooner the occurrence of multiple cracks in the thick layer is observed. The same applies for the matrix cracks nucleating in the thin layers. In all cases, a higher matrix crack density is observed in the thin layers in comparison to the thick layer as the test proceeds. Moreover, the crack rate is significantly higher in the thin 30° layers. It should be also mentioned that when comparing the [0°/30°]_2s_ laminates with the [0°/60°]_2s_ ones, noticeably lower densities are observed in the first case. The impact of the R-ratio was also in this case confirmed, with an R-ratio of 0.5 delaying the onset and propagation of damage.

**Figure 16:**
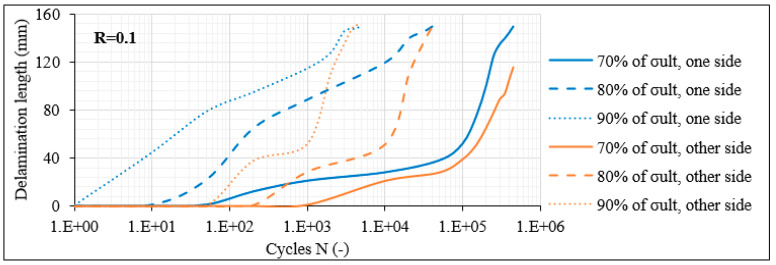
Delamination crack growth versus N in [0°/30°]_2s_ laminates for R = 0.1.

In Figure 16, the average delamination crack growth observed on both opposite external sides of the [0°/30°]_2s_ laminates for R = 0.1 is plotted. As a first remark, in the [0°/30°]_2s_ laminates, delaminations on both sides of the laminate were nucleated for all stress levels when R equalled 0.1, contrary to the [0°/60°]_2s_ laminates. This provides evidence for the detrimental influence of shear when fatigue loads are applied. This destructive behaviour is also confirmed when looking at the initiation period of delaminations. As an example, initial delaminations appeared in the [0°/30°]_2s_ laminates in the period 50–200 fatigue cycles for the 70% stress level for R = 0.1, whereas this occurred only in the period 10^3^–10^4^ cycles in the [0°/60°]_2s_ laminates for the same stress level. Similar behaviour was observed for R = 0.5. It should be mentioned that also the extent of damage was not the same in the two laminates. While in the [0°/30°]_2s_ specimens, delaminations propagated along the total gauge length in most cases, this did not always happen in the [0°/60°]_2s_ laminates.

Based on the damage observations discussed before, an empirical conclusion can be drawn regarding the [0°/30°]_2s_ laminates. Specifically, it was observed that in all test cases interlaminar delaminations were developed at the edge of the laminate when a certain matrix crack density in the middle thick layer was reached. This CDS was different for the two R-ratios under consideration. Specifically, it was noticed that for R = 0.1, interlaminar delaminations were detected at the laminate edge when the crack density reached a value in the range 0.1–0.2 cracks/mm in the middle thick layer. For the case of R = 0.5, delaminations were observed along the 0°/30° interface when the matrix crack density exceeded the value of 0.5 cracks/mm. In all cases, soon after the nucleation of delaminations, matrix cracks in both thin 30° layers were observed. However, it should be highlighted that the thin off-axis layers were not immediately cracked along the total gauge length. Initial matrix cracks appeared always close to the tip of the formed delamination in both off-axis layers and new ones kept on nucleating afterward along the layer length. Nevertheless, the total length was always cracked before delamination at the opposite laminate side was evident. Furthermore, it is notable that contrary to the [0°/60°]_2s_ laminates, the matrix crack density kept increasing after the nucleation and propagation of delaminations in the [0°/30°]_2s_ laminates. Therefore, the matrix cracking saturation criterion for the nucleation of delaminations does not apply for the last case. The above observations show that the matrix crack density and the damage conditions that suffice for leading to the initiation of delaminations in the [0°/30°]_2s_ laminates are much more limited compared to the [0°/60°]_2s_ laminates. A significantly lower CDS and therefore a considerably limited generated energy are enough for delaminations to appear due to the high shear stresses in the laminas. On the other hand, despite the fact that high matrix crack densities appear in the [0°/60°]_2s_ laminates, interlaminar delaminations are delayed, indicating a certain sensitivity of the composite material for the appearance of delaminations when shear is dominant.

**Figure 17:**
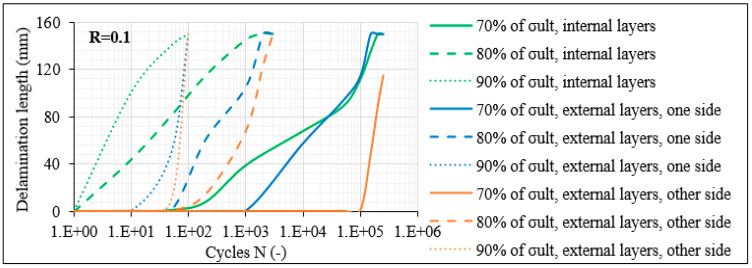
Delamination crack growth versus N in [0°/30°/0°/−30°]_s_ laminates for R = 0.1.

The beneficial response of the unbalanced laminates was also revealed for the specimens with 30° plies. In Figure 17, the measurements regarding the delamination lengths monitored in the [0°/30°/0°/−30°]_s_ specimens for R = 0.1 are reported. In this case, apart from the external (outer-ply) delaminations on both sides of the laminate (blue and orange curves), the length corresponding to internal (inner-ply) delaminations (green lines) is also reported. It is obvious that the balanced laminates are significantly more deteriorated in comparison to the unbalanced laminates for the same number of fatigue cycles and the same stress level. As an example, for the 80% σ_max_ and R = 0.1 test case, after 10^3^ fatigue cycles, three delaminations already appeared and propagated along almost the total gauge length in the [0°/30°/0°/−30°]_s_ laminates, whereas only two shorter delaminations developed in the unbalanced [0°/30°]_2s_ laminates. At the same time, it should be highlighted that fibre fracture always accompanied the inner-ply delaminations in the balanced laminates, leading to a further deterioration of the material. It should be noticed that in the balanced laminates, the CDS for the nucleation of delaminations corresponded to 0.05 cracks/mm for R = 0.1, revealing a lower fracture toughness than the unbalanced ones.

Regarding the [0°/30°/−30°]_2s_ laminates and the influence of the number of the off-axis layers on the fatigue response, unstable cracking phenomena were also in this case reported, such as in the 12-ply [0°/60°/−60°]_2s_ laminates. In this case, the deterioration of different regions was observed during testing, without however leading to coupling of the local delaminations along the total length. This fracture mode was observed only in these laminates, since in all previous cases, cracking was more uniform, initiating at one locus and propagating along the total specimen length. It was characteristic that in many cases, a full deterioration of one region was suddenly observed, consisting of multiple matrix cracks and delaminations along a limited length of maximum 10 mm. Due to the multiple interfaces along the laminate thickness, these fractured phenomena saturated almost immediately. Multiple fractured areas were observed along the laminate length as the test progressed, with a higher number when the maximum fatigue stress was increased. However, the fracture events in different laminate areas were independent for the longest time span of the test. As the test progressed, only the isolated outer-ply delaminations were finally connected, leading to a full-length propagation and final failure. Based on this description, the total deterioration of the 12-ply [0°/30°/−30°]_2s_ laminates was quite similar to that of the 8-ply [0°/30°/0°/−30°]_s_ laminates for the same fatigue conditions, a trend that was confirmed also under static loading [40].

#### 3.3.3. Edge Damage Microscopic Monitoring

**Figure 18:**
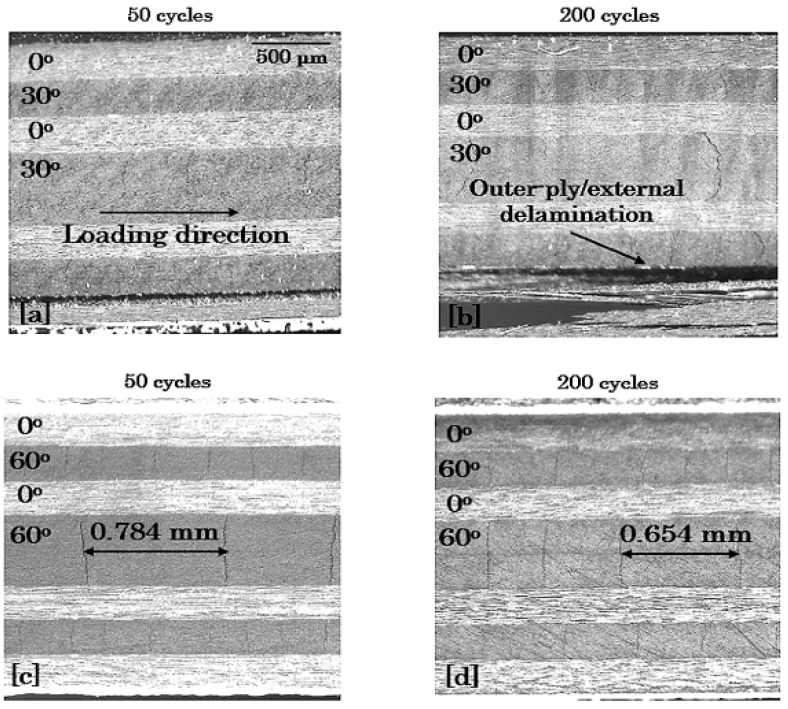
Damage in [0°/30°]_2s_ (top) and [0°/60°]_2s_ (bottom) laminates after (**a**,**c**) 50 and (**b**,**d**) 200 cycles for σ_max_ = 0.8 × σ_ult_ and R = 0.1.

**Figure 19:**
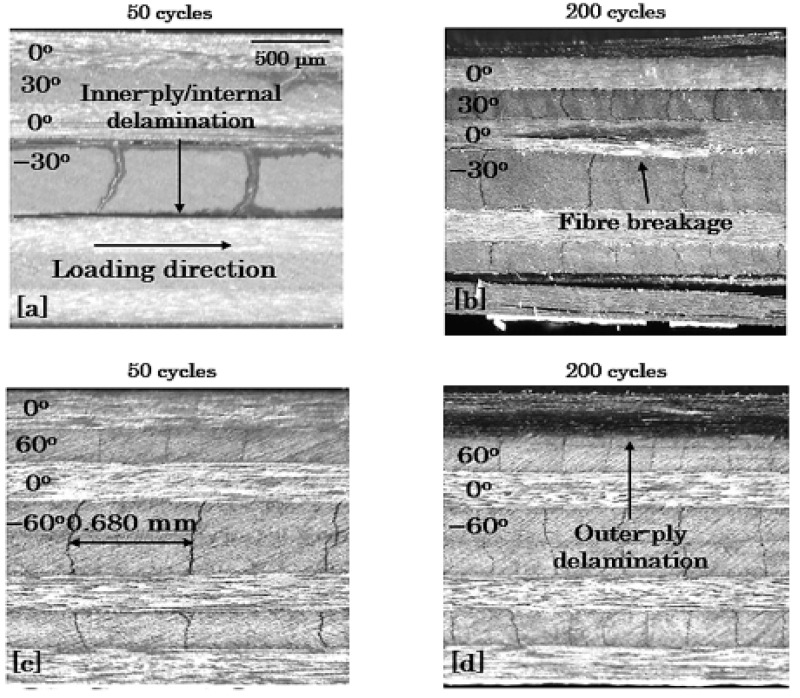
Damage in [0°/30°/0°/−30°]_s_ (top) and [0°/60°/0°/−60°]_s_ (bottom) laminates after (**a**,**c**) 50 and (**b**,**d**) 200 cycles for σ_max_ = 0.8 × σ_ult_ and R = 0.1.

**Figure 20:**
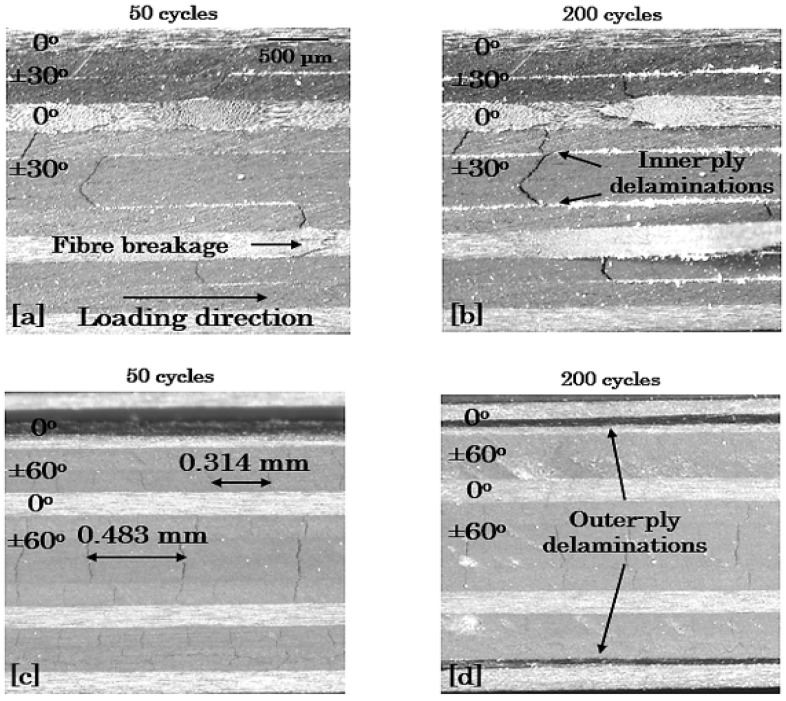
Damage in [0°/30°/−30°]_2s_ (top) and [0°/60°/−60°]_2s_ (bottom) laminates after (**a**,**c**) 50 and (**b**,**d**) 200 cycles for σ_max_ = 0.8 × σ_ult_ and R = 0.1.

To verify the previous discussion, characteristic microscopy images are shown in Figure 18, Figure 19 and Figure 20 for the 8-ply unbalanced and the 8-ply and 12-ply balanced lay-ups, respectively. To depict the aforementioned differences regarding the fatigue damage of the laminates, it was chosen to exhibit their damage state for σ_max_ = 0.8 × σ_ult_ and R = 0.1 after 50 and 200 cycles. All the findings obtained from the previous demonstration are supported by the microscopy images, which present both the qualitative and the quantitative damage state of the laminates. Delaminations are pronounced in the shear-dominated laminates and matrix cracking in the lay-ups with high σ_22_ stresses. The positive response of the unbalanced laminates is revealed, and the impact of the number of the off-axis plies is verified from the beginning of the fatigue life.

The previously presented damage findings at the edge of the laminates were also confirmed by performing midplane damage monitoring. To do this, specimens were loaded until a certain number of fatigue cycles, and then, they were cut along their middle axis. After polishing, damage monitoring was performed. The impact of the considered parameters was validated, confirming that the discussed trends are not only driven by edge phenomena.

### 3.4. Stiffness Degradation and Poisson’s Ratio Measurements

**Figure 21:**
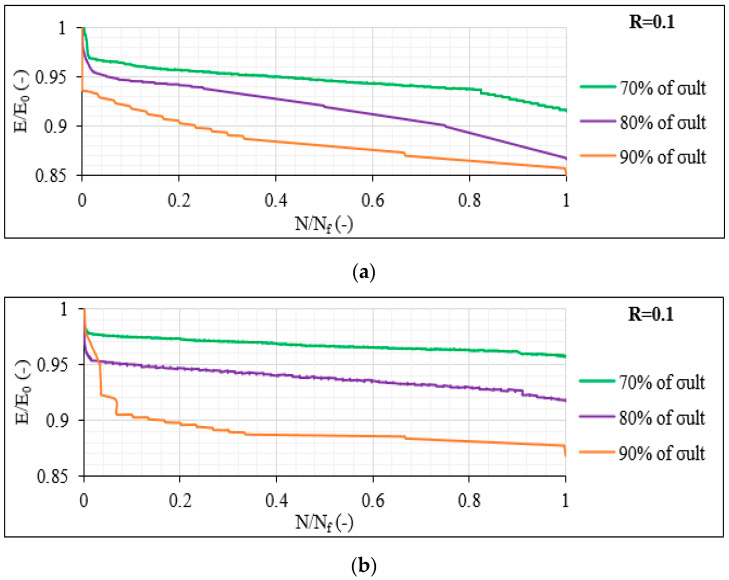
Stiffness degradation versus the normalised fatigue life in (**a**) [0°/30°]_2s_ and (**b**) [0°/60°]_2s_ laminates for R = 0.1.

All previous findings concerning the impact of shear, the comparison between balanced and unbalanced lay-ups and the influence of the number of off-axis layers were verified during the study of the stiffness degradation of the CFRP material. In Figure 21a,b, the measured stiffness degradation E/E_0_ (with E_0_ being the initial laminate stiffness) for representative [0°/30°]_2s_ and [0°/60°]_2s_ specimens is, respectively, plotted for R = 0.1 during the 70%, 80% and 90% σ_max_ cases versus the normalised fatigue life N/N_f_. It is obvious that a reduction in the stiffness is obtained when σ_max_ increases in both cases. A significant drop in stiffness is recorded during the first fatigue cycles, being higher for increasing σ_max_ levels. However, an importantly higher stiffness degradation is recorded for the [0°/30°]_2s_ samples during the fatigue tests.

**Figure 22:**
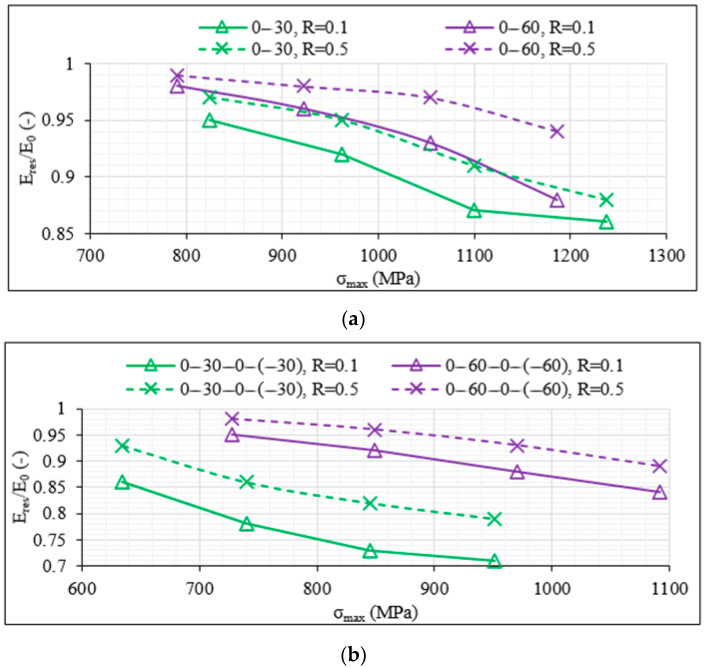
E_res_/E_0_ values versus σ_max_ obtained for (**a**) [0°/θ]_2s_ and (**b**) [0°/θ/0°/−θ]_s_ laminates.

To better display the impact of shear on the stiffness degradation as well as a comparison between the balanced and unbalanced laminates, in Figure 22a,b, the average E_res_/E_0_ results (where E_res_ is the residual stiffness of laminates that survived the run-out of one million cycles) for the 8-ply unbalanced and balanced laminates are, respectively, plotted. Shear seems to be in all cases detrimental. Matrix cracking in the transverse stress-dominated laminates leads to a significant drop in stiffness during the initial fatigue cycles, with, however, reduced decreasing rates as the test proceeds despite the high matrix crack densities. When shear is dominant, the stiffness degradation continues with a higher rate owing to the shear stresses and the forming delaminations, reducing the fracture toughness and leading to severe deterioration. The balanced laminates follow similar trends with, however, higher stiffness drops at lower stress levels when compared to the unbalanced lay-ups for the same fatigue conditions.

**Figure 23:**
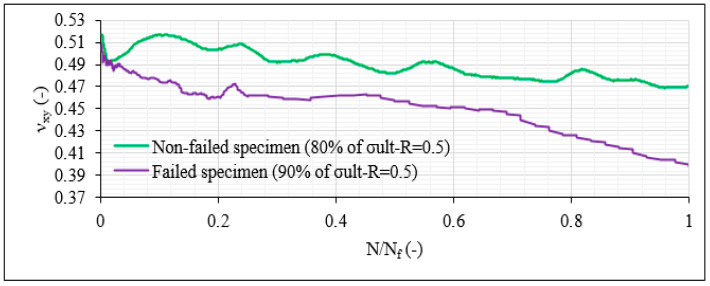
ν_xy_ evolution versus the normalised fatigue life for one nonfailed [0°/30°]_2s_ laminate (σ_max_ = 0.8 × σ_ult_ and R = 0.5) and one failed [0°/30°]_2s_ laminate (σ_max_ = 0.9 × σ_ult_ and R = 0.5).

After analysis of the Poisson’s ratio ν_xy_ (in the geometrical coordinate system) during the fatigue tests, it was revealed that it has the potential to be used for damage indications during the loading of the CFRP material. In most cases, the Poisson’s ratio had a decreasing trend over the total fatigue life, with that decrease always being higher for the more severe fatigue conditions, proving that the developing damage has an impact both on the longitudinal and the transverse strains, and therefore on ν_xy_. As an example, Figure 23 plots the ν_xy_ evolution versus the normalised fatigue life for one [0°/30°]_2s_ specimen subjected to the 80%-0.5 fatigue conditions and one specimen tested under σ_max_ = 0.9 × σ_ult_ and R = 0.5. The difference between the two samples lies in the fact that in the first case, the run-out of 10^6^ cycles was reached, while in the second case, the specimen failed after 425,693 cycles. In both cases, a continuous decrease in ν_xy_ is observed, although being lower in the case of the 80%-0.5 test and proving the sensitivity of the Poisson’s ratio to the developing damage in the material during the fatigue loading.

**Figure 24:**
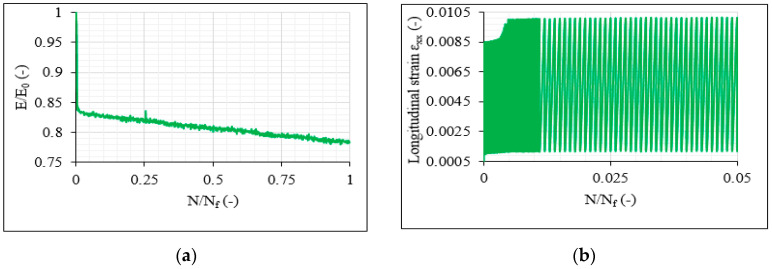
(**a**) Stiffness degradation versus the normalised fatigue life and (**b**) ε_xx_ evolution during the 0–0.05 N/N_f_ period for one [0°/30°/0°/−30°]_s_ laminate for the 70%-0.1 test.

As a final point, it should be mentioned that, in particular, the balanced [0°/30°/0°/−30°]_s_ and [0°/30°/−30°]_2s_ laminates presented a very characteristic response that should be better demonstrated. In these laminates, during the initial fatigue stage, a very sudden stiffness drop was always monitored with a continuous decrease thereafter, whereas in the unbalanced laminates, a more gradual stiffness decrease was observed. It was noticed that the higher the applied load and therefore the amount of developing damage, the higher this stiffness drop was. Moreover, for higher stresses, this drop in stiffness occurred earlier during the fatigue life, corresponding to the higher damage extent. To demonstrate this behaviour, Figure 24a plots the stiffness degradation of one 8-ply balanced [0°/30°/0°/−30°]_s_ laminate during the 70%-0.1 fatigue test. Contrary to the unbalanced laminates, a big drop in stiffness is suddenly observed during the initial fatigue cycles due to a rapid increase in the longitudinal ε_xx_ strains (in the direction of the loading), as shown in Figure 24b, plotting the ε_xx_ evolution for the same test from the beginning until 5% of N_f_. This response can be attributed to the generally higher deterioration of the balanced laminates, as already explained in the previous sections, with delaminations accompanied in all cases by fibre breakage. Another reason can be the faster propagation of the damage from the edge of the laminate toward the inner volume of the material, as confirmed by the mid-plane microscopic observations.

**Figure 25:**
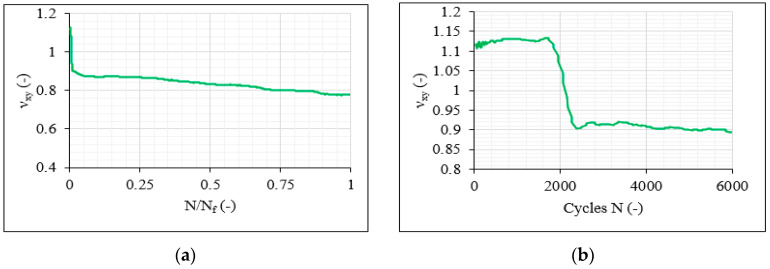
ν_xy_ evolution (**a**) versus the normalised fatigue life and (**b**) during the first fatigue 6 × 10^3^ cycles for one [0°/30°/0°/−30°]_s_ laminate for the 70%-0.1 test.

Following the observations regarding the stiffness degradation measurements, similar trends were also obtained for the Poisson’s ratio in the balanced laminates. A characteristic example is presented in Figure 25a for the same balanced [0°/30°/0°/−30°]_s_ laminate tested under the 70%-0.1 fatigue conditions. A decreasing trend is also in this case recorded with a significant drop after a certain amount of fatigue cycles. It was generally observed that in the more severe fatigue conditions, a higher drop of the Poisson’s ratio occurred, appearing earlier during the fatigue life and being always accompanied by a more gradual decrease afterward. Specimens tested under lower stress levels and reaching a higher number of fatigue cycles were characterised by a small increasing tendency of ν_xy_ during the initial fatigue cycles, as shown in Figure 25a, followed later by a sudden drop, attributed to rapid increase in the longitudinal ε_xx_ strains and decrease in the transverse ε_yy_ strains due to the propagation of the damage toward the inner volume of the laminate. In Figure 25b, the evolution of the Poisson’s ratio during the first 6 × 10^3^ fatigue cycles is plotted for the same specimen to demonstrate the above description.

## 4. Conclusions and Observations

- The effect of multiaxiality, a comparison between balanced and unbalanced composite laminates and the impact of the number of off-axis layers on the tension–tension fatigue response were demonstrated in this work by testing angle-ply CFRP [0°/θ]_2s_, [0°/θ/0°/−θ]_s_ and [0°/θ/−θ]_2s_ laminates for two different off-axis angles, namely 30° and 60°.
- The mechanical response was initially studied in terms of S-N_f_ and residual strength data. The impact of shear was clearly indicated with laminates consisting of 30° off-axis plies leading to lower N_f_ and residual strength values.
- A high impact of the R-ratio was also revealed. An R-ratio equal to 0.5, corresponding to lower stress amplitude during fatigue loading, resulted always in higher fatigue life.
- One of the most interesting findings concerns the response of the unbalanced laminates when compared with the balanced lay-ups. Both in terms of S-N_f_ and residual strength data, a much better performance was observed for the first time for the unbalanced laminates having the same off-axis angle and being tested under the same fatigue conditions as the balanced ones.
- All findings were confirmed by studying the progressive damage accumulation during fatigue. In all cases, matrix cracking was the initial damage mode appearing, leading to the interlaminar delaminations and final fibre breakage in laminates that did not survive the run-out of one million cycles. Nevertheless, important uncoverings were obtained when the shear-dominated laminates were compared with the laminates in which the transverse stresses dominate the stress state.
- Specifically, for the laminates with 60° off-axis plies, matrix cracks appeared in all off-axis layers during the very first fatigue cycles. Moreover, a saturation criterion applied, indicating that when the transverse stresses determine the damage accumulation, interlaminar delaminations during fatigue loading occur only after a certain saturation of the matrix cracks was reached.
- It was confirmed that the CDS was very similar both for the balanced and the unbalanced 8-ply laminates. However, it was reached quite earlier in the balanced lay-up, leading more quickly to the initiation and propagation of interlaminar delaminations for the same stress level.
- Significantly higher matrix crack densities were acquired though for the 12-ply laminates with an off-axis angle of 60°, showing a certain impact of the number of off-axis plies on the fatigue response.
- By contrast, the damage response was different in the shear-dominated laminates. In addition, in this case, matrix cracking was the initial damage mode appearing. However, initial matrix cracks only appeared in the middle thick layer in the 8-ply balanced and unbalanced lay-ups, and the onset of cracking was not consistently observed during the very first fatigue cycles. Only when the crack density reached a certain value in the middle thick layer, delaminations and matrix cracks in the thin off-axis layers initiated.
- The crack density limit, after which the delaminations started occurring in the laminate was quite smaller for the balanced laminates, indicating a significantly lower fracture toughness.
- The 12-ply shear-dominated laminates presented a somehow different damage response. In this case, the multiple interphases owing to the higher number of off-axis layers rapidly led to the local deterioration of the laminate at certain fractured areas. Nevertheless, the interphases acted also as obstacles for the development of damage along the total laminate length.
- The impact of the different parameters on the fatigue response was confirmed in all cases by applying both edge and midplane microscopy.
- Finally, stiffness degradation and Poisson’s ratio measurements validated the response of the material under the different conditions.
- Higher stiffness degradation was observed for increasing shear stresses and for the balanced lay-ups.
- The Poisson’s ratio seemed to be a satisfactory qualitative damage indicator, maintaining reducing trends during fatigue and correlating promisingly with the extent of damage.

## Figures and Tables

**Table 1:** Measured mechanical properties of the Carbon Fibre Reinforced Polymer (CFRP) material.

Property	Value (Average and Standard Error)	Unit
σ_11,ult_	2272 ± 89	MPa
σ_22,ult_	53 ± 2	MPa
τ_12,ult_	52 ± 1	MPa
E_11_	125.8 ± 3.9	GPa
E_22_	9.4 ± 0.3	GPa
G_12_	4.1 ± 0.1	GPa
ν_12_	0.335 ± 0.014	-

**Table 2:** Frequency of microscopic inspection for the fatigue tests.

**Fatigue Tests**	**Microscopic Inspection after (x) Cycles**	
For N_f_ = 10^6^ cycles	10, 50, 200, 10^3^, 10^4^, 10^5^, 3 × 10^5^, 5 × 10^5^, 7.5 × 10^5^, 10^6^	
**Condition**	**Cycle count**	**Microscopic inspection every (x) cycles**
If N_f_ < 10^3^	50-failure	50
If N_f_ < 10^4^	10^3^-failure	10^3^
If N_f_ < 10^5^	10^4^-failure	10^4^
If N_f_ < 10^6^	5 × 10^4^-failure	5 × 10^4^

**Table 3:** Digital Image Correlation (DIC) system configuration.

Parameter	Value	Unit
Lenses	23	mm
Subset size	21	pixels
Step size	7	pixels
Resolution (x × y)	2085 × 896	pixels
Sensitivity	0.028	mm/pixel
Pixel resolution (x/y)	28/28	μm/μm
Subset resolution (x/y)	588/588	μm/μm
Average speckle diameter	100	μm

**Table 4:** DIC image acquisition frequency for the fatigue tests.

Cycle Count	Image Taken Every (x) Cycles
0–500	20
500–10^3^	50
10^3^–10^4^	200
10^4^-N	10^3^

## Data Availability

Data sharing is not applicable to this article.
